# Cells of the Innate and Adaptive Immune Systems in Kaposi's Sarcoma

**DOI:** 10.1155/2020/8852221

**Published:** 2020-11-21

**Authors:** Owen Ngalamika, Sody Munsaka

**Affiliations:** ^1^Dermatology and Venereology Division, University Teaching Hospital, University of Zambia School of Medicine, Lusaka, Zambia; ^2^Department of Biomedical Sciences, School of Health Sciences, University of Zambia, Lusaka, Zambia

## Abstract

Kaposi's sarcoma (KS) is an angioproliferative malignancy whose associated etiologic agent is the Kaposi's sarcoma-associated herpesvirus (KSHV). KS is the most prevalent malignancy among HIV-infected individuals globally and is considered an AIDS-defining malignancy. The different forms of KS including HIV-associated KS, iatrogenic (immunosuppression-related) KS, and classical KS in elderly males suggest that immune cell dysregulation is among the key components in promoting KS development in KSHV-infected individuals. It is therefore expected that different cell types of the immune system likely play distinct roles in promoting or inhibiting KS development. This narrative review is focused on discussing cells of the innate and adaptive immune systems in KSHV infection and KS pathogenesis, including how these cells can be useful in the control of KSHV infection and treatment of KS.

## 1. Introduction

Kaposi's sarcoma (KS) is a vascular malignancy that is highly prevalent among HIV-infected individuals. KS causes significant morbidity and mortality in sub-Saharan Africa where seroprevalence of both HIV and the Kaposi's sarcoma-associated herpesvirus (KSHV) are high [1, 2]. Many factors have been reported to predispose KS development and progression. However, immune dysregulation appears to be a common factor in all forms of KS development. For instance, HIV-associated (epidemic) KS is usually a consequence of severe immunocompromise [3], Iatrogenic KS is a result of immunosuppressive therapy [4], while Classical KS affects elderly men [5] who likely have compromised immunity due to aging [6, 7].

Kaposi's sarcoma-associated herpesvirus (KSHV) also known as Human Herpesvirus type 8 (HHV-8) is the etiologic agent associated with the development of all types of KS and is found in all KS lesions [8]. It has been observed to establish latency and encode proteins that compromise immune surveillance leading to lifelong infection [9, 10]. Utilizing and building on existing evidence on the defects in immunity that predispose to KSHV infection or replication and KS tumor development will enhance the fight against KS by providing mechanisms that may be targeted for immune therapy or vaccine development.

Both innate and adaptive immune responses have been extensively studied in KSHV infection and KS. KSHV is known to modulate cellular genes to control innate immune responses against primary infection [11]. Adaptive immunity, particularly cell-mediated immunity, seems to be more paramount in fighting viral infections such as KSHV and malignancies such as KS [12]. On the other hand, humoral immunity may also be a key component in producing antibodies involved in neutralizing viruses, opsonization, complement activation, and development of antibody-dependent cellular cytotoxicity (ADCC) against malignancies. For instance, anti-KSHV neutralizing antibodies are present in active KS disease, though these antibodies have been found to have no role in protection against KSHV infection and KS [13]. This narrative review aims to explore innate and adaptive immune responses with a focus on cells of the innate and cellular adaptive immune system in KSHV infection and KS. Exploring these cells is important, because they express proteins that are important in signaling pathways that lead to effector functions that ultimately result in fighting off infections and cancer. In addition, some of the cells of the immune system may be permissive to infections by microorganisms such as KSHV, and may harbor KSHV, and hence provide insights on how KSHV evades immune surveillance and establishes latency. This review also discusses on how cells of the immune system can be a target in control of KSHV infection and in KS treatment.

## 2. Methods

We searched the literature using pubmed and google scholar using the following keywords: Kaposi's sarcoma, Kaposi's sarcoma-associated herpesvirus, mast cells, eosinophils, neutrophils, monocytes, macrophages, Langerhans cells, natural killer cells, CD4^+^ T cells, T helper 1 cells, T helper 2 cells, T helper 17 cells, regulatory T cells, CD8^+^ T cells, innate immunity, and adaptive immunity. We selected articles that were most recent, preferably in the last 5 years, but also included very relevant older articles that had strong evidence to support the statements reflecting cells of the immune system in inhibiting or promoting KSHV infection and KS development. We focused on original articles but also included some review articles.

## 3. Cells of Innate Immunity in KSHV Infection and KS

### 3.1. Mast Cells

Mast cells (MCs) are tissue-resident cells that arise from bone marrow-derived progenitor cells. They constitutively express the immunoglobulin E (IgE) receptor [14]. MCs are commonly associated with anaphylaxis and immediate hypersensitivity reactions [15, 16]. However, MCs have also been reported to be associated with KSHV infection and KS tumorigenesis [17]. In one study, MCs were identified as KSHV reservoirs that support latent and lytic KSHV infection [18]. From this study, MCs were also observed to be closely associated with KS spindle cells. In addition, treatment with MC antagonists resulted in a rapid and durable regression of HIV-associated KS lesions [18]. Another study observed and reported that immunoglobulin E, a potent activator of MCs, was associated with the presence and severity of KS [17]. Interestingly, a more recent study found that histamine (one of the major substances released by MCs), through the MAPK and PI3K/Akt signaling pathways, promotes KSHV lytic replication, and knockdown of histamine receptors blocks KSHV lytic replication [19]. From these studies, it appears that mast cells and their product histamine may be a potential target in the study and control of KSHV infection and KS.

### 3.2. Eosinophils

Eosinophils are white blood cells that contain acidophilic granules. They are proinflammatory cells involved in allergic reactions such as asthma and in combating multicellular parasites [20]. Eosinophils are known to be elevated in parasitic infestations [21]. The role of eosinophils in KSHV infection and KS is yet to be established. However, an association between KSHV seropositivity with helminth infestations and malaria has been previously observed and reported [22]. Other studies have found associations between active KS disease with parasitic infestations [23]. Further studies are required on the role of parasitic infestations and eosinophils in KSHV infection and KS pathogenesis.

### 3.3. Neutrophils

Neutrophils make up a majority of all white blood cells in the body. Through phagocytosis, release of cytokines/chemokines, and release of reactive oxygen species, neutrophils play a key role in innate immune responses to infections in the body. It has been observed that KSHV encodes a protein (vOX2) that inhibits neutrophil function by suppressing the oxidative burst and inhibition of production of proinflammatory chemokines [24]. In addition, KSHV infection of endothelial cells has been shown to inhibit neutrophil recruitment via an IL-6-dependent mechanism [25]. Here, KSHV infection is associated with an increase in IL-6 expression which results in increased expression of the suppressor of cytokine signaling 3 (SOCS3). SOCS3 is associated with inhibition of neutrophil recruitment to areas of inflammation by inhibition of mediators such as IL-1 and TNF*α* [26]. In HIV-associated KS patients, neutrophil function has been reported to be compromised [27]. This is supported by a recent study on tumor infiltrating immune cells in KS tumors, which did not observe any neutrophil infiltration in KS tumor biopsies [28]. Therefore, the KS tumor environment and associated cytokines which are elevated in KS patients, such as IL-6, may also contribute towards KS immune evasion through the neutrophil pathway. It is highly likely that neutrophils may play a significant role in controlling KSHV infection and KS.

### 3.4. Monocytes and Macrophages

Monocytes are the largest in size among all white blood cells and are the precursors of tissue macrophages. Macrophages are responsible for detecting, engulfing, and destroying extracellular pathogens [29]. Monocytes and macrophages appear to promote KSHV infection and KS development [30, 31]. It has been reported previously that infection of endothelial cells by KSHV induces the expression of cytokines including IL-6, IL-10, IL-13, and angiopoietin-2 that drive monocytes to differentiate and polarize into tumor-associated macrophages (TAMs) [32]. These TAMs are M2 macrophages that express the unique surface markers CD163, CD206, and legumain; they are known to promote angiogenesis and suppress antitumor immunity and therefore contribute towards tumor development and progression [33, 34]. It has also been previously observed that latent infection of monocytes by KSHV results in downregulated expression of cytokines and costimulatory receptors of the adaptive immune response [35]. This is also supported by a study that identified monocytes as a reservoir for KSHV in KS tumors [36]. Furthermore, macrophages have also been observed to be evenly distributed and in high numbers in KS tumors [28] and may provide a reservoir of KSHV.

### 3.5. Langerhans Cells

Langerhans cells are dendritic cells of the epidermis involved in immune surveillance. They are involved in both innate and adaptive immune responses [37]. Studies have observed and reported that KSHV infects Langerhans cells and other dendritic cells of the skin and mucosa (monocyte-derived dendritic cells and interstitial dermal dendritic cells) [38]. In Langerhans cells and interstitial dermal dendritic cells, KSHV is able to undergo full lytic replication and impairs the ability of these cells to stimulate CD4^+^ helper T cell responses [38]. However, despite KSHV seemingly attempting to impair dendritic cell function, there has been evidence suggesting that dendritic cells from patients with HIV-/AIDS-associated KS retain the ability to prime CD8^+^ T lymphocytes against KSHV-specific antigens [39]. On the other hand, in AIDS-associated KS, Langerhans cells have been observed to be significantly reduced in number in both oral and skin KS lesions [40, 41]. This suggests that Langerhans cells may play a positive role in immunity against KSHV infection and KS.

### 3.6. Natural Killer Cells

Natural killer (NK) cells are lymphocytes of the innate immune system that recognize and kill virally-infected cells and neoplastic cells [42]. NK cells function by utilizing activating or inhibitory receptors that distinguish between normal cells and abnormal cells that have been altered through infection or other means such as neoplasia [43]. Two main distinct ways that NK cells recognize and kill infected or cancerous cells are natural killing of cells with downregulated major histocompatibility complex (MHC) class I molecules on their cell surfaces [44], and by antibody-dependent cellular cytotoxicity (ADCC) through the IgG receptor (CD16) which enables recognition and killing of cells opsonized with antibodies [45, 46]. What role NK cells play during KSHV infection and KS development is not clear. It has been observed that KSHV inhibits NK cell-mediated cytotoxicity by expressing a protein (K5) that downregulates ICAM-1 and B7-2, which are ligands for NK cell-mediated cytotoxicity receptors [47]. In addition, KSHV expression of K5 downregulates the ligands for NK activating receptors (NKG2D and NKp80) thereby providing a mechanism to evade NK cell antiviral activity [48, 49]. It appears that increasing the NK cell functional recognition of KSHV-infected cells may be a strategy that can be adopted in the fight against KS.

## 4. Adaptive Responses in KSHV Infection and KS

### 4.1. CD4^+^ T Cells

#### 4.1.1. T Helper 1 Cells

T helper 1 (Th1) cells are a phenotype of CD4^+^ helper T cells that promote cell-mediated immunity against intracellular microorganisms. Among the main effector cells for Th1 immunity are macrophages and cytotoxic T lymphocytes [50, 51]. Key cytokines produced by Th1 cells include IL-2, TNF-*α*/*β*, and IFN-*γ*/*β* [52, 53]. Suppression of type 1 IFNs is associated with KSHV lytic replication [54]. Some studies have observed that KSHV evades antiviral immunity by encoding the chemokine ligand, vCCL2, that antagonizes the chemokine receptors CCR1 and CCR5 which are expressed on Th1 lymphocytes [55, 56]. Overall, KSHV promotes a Th2 response which is important in fostering a humoral response and inhibits a Th1 response, as supported by studies that have demonstrated that CD4^+^ T cells from KS tumors produce IL-4 rather than IFN-*γ* [57]. IL-4 promotes differentiation to Th2 cells and is expressed by Th2 cells to promote the proliferation of B cells, production of antibodies, and immunoglobulin class-switching [58].

#### 4.1.2. T Helper 2 Cells

T helper 2 (Th2) cells are a phenotype of CD4^+^ helper T cells that promote humoral immunity, particularly against extracellular parasites [59]. In HIV infection, there has been observed a decline in Th1 activity and an increase in Th2 activity [60]. KSHV infection of dendritic cells has been reported to intentionally induce a skewing towards a Th2 response [61]. KSHV has also been observed to encode the chemokine ligands (vCCL1, vCCL2, and vCCL3) that bind to chemokine receptors (CCR8, CCR3, and CCR4) expressed on the surface of Th2 cells and hence result in chemoattraction of Th2 lymphocytes to infection sites [62]. This may explain the observed rise in anti-KSHV antibodies in KS patients [63]. However, there appears to be no role of these neutralizing antibodies (nAb) in controlling KSHV infection or KS [63]. The high KSHV nAb in KS patients may be attributed to persistent production of antibody-associated cytokines (IL-4 and IL-5) and not necessarily the control of KSHV infection or KS [63, 64].

#### 4.1.3. T Helper 17 Cells

T helper 17 (Th17) cells are proinflammatory cells characterized by IL17 production and protection against extracellular pathogens including bacteria and fungi [65]. IL17 is a key cytokine that promotes neutrophil mobilization and inflammation and is associated with autoimmune diseases including psoriasis and rheumatoid arthritis [66, 67]. Th17 cells do not appear to have a role in KS disease or pathogenesis as evidenced from studies that have shown that IL17 production is not differential between KS patients and KSHV-infected non-KS individuals [68].

#### 4.1.4. Regulatory T Cells

Regulatory T cells (Tregs) are a subtype of T cells that modulate the immune system by suppressing the proliferation of effector T cells and thereby prevent autoimmune diseases [69, 70]. In cancer, Tregs have been observed to be upregulated in number and function and thereby promote tumorigenesis by inhibiting immune responses against malignancies [71]. In KS, some studies have reported that Tregs are present in KS tumors and that they are markedly increased in most advanced KS lesions [72]. However, in another study, a significant increase in proportion of Tregs was observed in KSHV-infected individuals who developed KS compared to those who did not [73]. Therefore, the role of Tregs in regulating tumorigenesis will need further study to determine whether it could be used as potential target in controlling KS.

### 4.2. CD8^+^ T Cells

CD8^+^ T cells, also known as cytotoxic T lymphocytes (CTL), are primarily responsible for killing cancer cells and cells infected with viruses [74]. CTLs recognize antigens presented by MHC class 1 molecules [75]. Upon recognition of specific MHC-peptide complexes in secondary lymphoid organs, naive CTLs differentiate into effector cells that migrate to affected sites to clear infection or tumors [76]. CTLs kill target cells by releasing the cytotoxic proteins granzyme and perforin, inducing apoptosis through the Fas-Fas ligand interaction, and release of cytokines that trigger targeted cell death [77].

In KS, there has been reported a poor localization of CTLs in KS tumors. In one study, it was observed that KSHV-specific CTLs were not recruited to KS lesions in KSHV-infected individuals who progressed to KS [78]. This is supported by a more recent study that observed and reported statistically significant sparse distribution of CD8^+^ T cells in regions of KS tumor biopsies with evident KSHV-infected cells compared to regions of KS tumor biopsies that were devoid of KSHV infection which had readily detectable CD8^+^ T cells [28].

Functionality of CTLs against KSHV peptides in KS patients and KSHV-infected but KS-asymptomatic individuals has also been studied [78, 79]. KSHV-specific T cells have been found to be significantly less in HIV-associated and non-HIV-associated KS patients than in KSHV-infected individuals without KS [79]. In another study, it was found and reported that KSHV-specific CTLs were rare in KSHV-infected individuals who progressed to KS, while they were more abundant in KSHV-infected individuals who controlled the infection and did not progress to KS [78]. It has also been observed that KSHV encodes viral OX2, which is an orthologue of cellular CD200 that attenuates antigen-specific T cell responses [80].

CD8^+^ T cell responses against KSHV have been observed to be towards proteins expressed in the lytic phase as opposed to the latent phase of the viral life cycle [81]. The CD8^+^ T cell-recognized proteins are encoded by KSHV early lytic gene pools including ORF8/ORF49/ORF61 and ORF59/ORF65/K4.1, and the KSHV late lytic gene pool ORF28/ORF36/ORF37 [81]. These CD8^+^ T cell responses against KSHV have been found to be weak in both KSHV seropositive individuals without KS and in KS patients [82]. In addition, there is a lack of immunodominance of CD8^+^ T cell responses towards KSHV antigens as evidenced by variable specificities towards KSHV epitopes, with diverse and sparse responses in both healthy KSHV seropositive individuals and KS patients [82].

CD8^+^ T cells that are polyfunctional by producing more than one immune mediator (such as IFN*γ*, MIP-1*β*, and TNF*α* and/or CD107) have been associated with superior control of lasting viral infections such as HIV-1 [83]. These polyfunctional CD8^+^ T cells have been observed in KSHV seropositive individuals who do not have KS [84]. However, it has also been observed that disappearance of polyfunctional CTLs is associated with virologic reactivation of a human herpesvirus, Epstein bar virus (EBV) [85]. This existing evidence suggests that CTLs are important but ineffective in the control of KSHV infection, especially in individuals who develop KS. This may be due to poor localization of CTLs in KS tumors and hence reduced exposure to KSHV antigens, expression of proteins by KSHV that result in evasion of the immune system, or poor immunodominance or weak immune responses upon encounter of KSHV antigens by CTLs.

### 4.3. B Cells

B cells are an important part of the adaptive immune system that act both as antigen-presenting cells and produce antibodies [86, 87]. Upon activation, B cells differentiate into plasma cells that produce antibodies to specific antigens at a larger scale [88]. KSHV infects CD19+ B cells, with a majority of infected cells expressing immunoglobulin M [89]. It has also been observed that the latency-associated nuclear antigen (LANA) limits efficient CD8+ T cell recognition of KSHV-infected B lymphocytes, while LANA-specific CD4+ T cells are important effectors in the control of KSHV infection of these cells [89, 90].

Anti-KSHV neutralizing antibodies are known to be high in KSHV-infected individuals with active KS disease, while they are low or undetected in KSHV-infected individuals without KS [13]. Studies have reported that KSHV infection induces maturation of naive B cells resulting in antibody production [89, 91]. It seems that anti-KSHV neutralizing antibodies have little or no role in the control of KSHV infection and KS.

## 5. Immunomodulators in KS Treatment

Since the pathogenesis of KS is often associated with a dysregulated immune system, immunomodulators are actively studied as targeted treatment for KS. Some immunomodulatory therapies against KS have been disappointing, while others have shown promise [92]. For instance, bevacizumab (an anti-VEGF monoclonal antibody) has a low response rate in KS treatment, despite VEGF being a growth factor for KS cells [93]. Imatinib (a c-kit/PDGF-receptor inhibitor) has shown activity against AIDS-KS, but response rates have also been low [94]. Interaction between the programmed cell death protein 1 (PD1), an immune checkpoint expressed on CD8^+^ cytotoxic T lymphocytes, and its ligand PD-L1 expressed on cancer cells leads to inhibition of cancer-specific immune responses [95, 96]. Trials on immune checkpoint inhibitors against PD1 and PD-L1 for treatment of KS are promising but still inconclusive [97].

Pomalidomide has been the most effective immunomodulatory agent so far against KS [98]. Pomalidomide is a derivative of thalidomide that has antiangioproliferative, immunomodulatory, and antiangiogenic effects [99]. In KS, pomalidomide therapy has been associated with a rapid favourable response, with more than 70 percent of individuals responding to treatment, regardless of HIV status and disease stage [98]. This response to pomalidomide was correlated with an increase in CD4^+^ and CD8^+^ T cells in both HIV-associated KS patients and KS patients without HIV infection [98]. Since cell-mediated immunity is important for KS and KSHV control, with poor cell-mediated immunity predisposing to KS development and progression, it appears that the pomalidomide effect may be through enhancing the cell-mediated immunity in KS patients.

## 6. Conclusion

Cells of both the innate and adaptive immune systems are involved in promoting or controlling KSHV infection and KS disease. Mast cells harbor KSHV and also promote KSHV lytic replication through histamine release and are a potential therapeutic target for control of KSHV infection and KS. Treatment with agents that inhibit mast cells or their product histamine is a potential therapeutic approach for KS. Monocyte/macrophages harbor the KSHV virus and can be a target for control of KSHV infection, while Tregs are upregulated in KS and inhibit immune responses against KS and KSHV. Use of anti-IL-6 therapy may be a potential therapeutic strategy to promote neutrophil chemotaxis into KS lesions and thereby enhance the control of KSHV infection and KS. NK cells appear to be important in innate immunity against KS. CD8^+^ T cells are important but ineffective against the control of KSHV infection and KS. Immunomodulators such as pomalidomide that increase the CD4^+^ and CD8^+^ T cells are an effective therapeutic option for KS.
